# Derivation of a risk-adjusted model to predict antibiotic prescribing among hospitalists in an academic healthcare network

**DOI:** 10.1017/ash.2024.422

**Published:** 2024-10-07

**Authors:** Udodirim N. Onwubiko, Christina Mehta, Zanthia Wiley, Jesse T. Jacob, K. Ashley Jones, Julianne Kubes, Hasan F. Shabbir, Sujit Suchindran, Scott K. Fridkin

**Affiliations:** 1 Department of Epidemiology, Rollins School of Public Health, Emory University, Atlanta, GA, USA; 2 Department of Medicine, Division of Infectious Diseases, Emory University School of Medicine, Atlanta, GA, USA; 3 Department of Pharmacy, Emory Healthcare, Atlanta, GA, USA; 4 Office of Quality, Emory Healthcare, Atlanta, GA, USA

## Abstract

**Background:**

Among inpatients, peer-comparison of prescribing metrics is challenging due to variation in patient-mix and prescribing by multiple providers daily. We established risk-adjusted provider-specific antibiotic prescribing metrics to allow peer-comparisons among hospitalists.

**Methods:**

Using clinical and billing data from inpatient encounters discharged from the Hospital Medicine Service between January 2020 through June 2021 at four acute care hospitals, we calculated bimonthly (every two months) days of therapy (DOT) for antibiotics attributed to specific providers based on patient billing dates. Ten patient-mix characteristics, including demographics, infectious disease diagnoses, and noninfectious comorbidities were considered as potential predictors of antibiotic prescribing. Using linear mixed models, we identified risk-adjusted models predicting the prescribing of three antibiotic groups: broad spectrum hospital-onset (BSHO), broad-spectrum community-acquired (BSCA), and anti-methicillin-resistant Staphylococcus aureus (Anti-MRSA) antibiotics. Provider-specific observed-to-expected ratios (OERs) were calculated to describe provider-level antibiotic prescribing trends over time.

**Results:**

Predictors of antibiotic prescribing varied for the three antibiotic groups across the four hospitals, commonly selected predictors included sepsis, COVID-19, pneumonia, urinary tract infection, malignancy, and age >65 years. OERs varied within each hospital, with medians of approximately 1 and a 75th percentile of approximately 1.25. The median OER demonstrated a downward trend for the Anti-MRSA group at two hospitals but remained relatively stable elsewhere. Instances of heightened antibiotic prescribing (OER >1.25) were identified in approximately 25% of the observed time-points across all four hospitals.

**Conclusion:**

Our findings indicate provider-specific benchmarking among inpatient providers is achievable and has potential utility as a valuable tool for inpatient stewardship efforts.

## Introduction

Antibiotic resistance (AR) poses a significant public health concern in the United States (US), with an estimated three million cases of antibiotic-resistant (AR) infections occurring annually, leading to approximately 49,000 deaths.^1^ A key driver of the emergence of AR infections is the unnecessary use of antibiotics in healthcare delivery,^1,2^) which contributes to prolonged hospital stays, increased clinic visits, and higher incidence of long-term disability.^3–5^ Among hospitalized patients, suboptimal antibiotic prescribing occurs when antibiotics are initiated without supporting microbiologic or clinical evidence or when they are continued beyond recommended durations.^6^ Prior studies indicate that 30%–50% of inpatient antibiotic orders involve incorrect choice of agent, treatment duration, or application for the indicated treatment.^6,7^

Various tools exist for performing antibiotic stewardship activities in healthcare, including reporting location-specific rates of antibiotic transactions to the Centers for Disease Control and Prevention (CDC) National Healthcare Safety Network (NHSN), which provides a site-specific metric for usage comparisons.^8–10^ While these metrics are valuable for monitoring usage, they have limitations that include inadequate risk adjustment in the calculated metrics and limited ability to attribute poor prescribing to specific prescribers.^11,12^ In inpatient settings, where patient care involves a team of rotating clinicians, attributing the choice or duration of any single antibiotic during a patient’s hospital stay to a specific provider is challenging and often inaccurate. The lack of standardized metrics for comparing prescriber-level prescribing limits accurate provision of feedback to individuals, a proven tool for inducing behavioral changes and reducing inappropriate prescribing in experimental studies conducted in nonacute care.^13–16^

Peer comparison of providers is most effectively accomplished through risk adjustment, typically achieved by comparing each provider’s observed prescribing activity against the expected activity considering patient population characteristics. In some outpatient studies, no adjustment is needed, such as when focusing on respiratory infection in pediatric patients, while in others, adjusting for variables such as age or diagnosis has proven useful.^17,18^ This study takes advantage of a large healthcare network where clinical data from four hospitals are archived in a structured system for research purposes that allows accurate attribution of specific dates of antibiotic prescribing to individual hospitalists. Additionally, we leveraged the clinical data to account for diverse patient comorbidities, attempting to adjust for varying patient conditions that may influence antibiotic prescribing across providers. Our study’s objectives include deriving a patient risk-adjusted metric for comparing antibiotic prescribing among hospitalists and using this metric to highlight patterns of antibiotic use among providers at the four hospitals examined. While this paper does not systematically evaluate the feasibility of deriving this metric in other hospitals, the intent of developing this metric is to later evaluate the benefits of using this metric for provider-specific feedback in a clinical trial to reduce unnecessary antibiotic use.

## Methods

### Data source

Data covering the period from January 2020 to June 2021 were extracted on inpatients from each of four hospitals in our healthcare network where Hospital Medicine Services are provided. Hospitals A and B were 582-bed and 537-bed academic medical centers, respectively, while hospitals C and D were 373-bed and 125-bed community hospitals, respectively (Supplemental Table S1). Staffing models were mostly similar at each with 2–4 Nocturnists at each facility, but Advanced Practice Providers worked only at the two larger facilities. For each hospitalist in each hospital, we utilized billing data to identify patient encounters for which the hospitalist provided care and calculated the number of days each provider cared for each patient (billed patient days, bPD). All hospitals have a robust antibiotic stewardship team in place with harmonization of clinical practice guidelines and effort across all facilities.

### Days of therapy

We extracted antibiotic prescribing data from the electronic medication administration records (eMAR), which accounts for returned medications and captures only administered medications. For each bPD, matching transaction dates in the eMAR with an order for antibacterial agents belonging to any of the three NHSN antibiotic groups—broad spectrum hospital-onset (BSHO), broad-spectrum community-acquired (BSCA), and anti-methicillin-resistant *Staphylococcus aureus* (Anti-MRSA)—were captured as billed days of antibiotic therapy (bDOT). bDOT differs from DOT in that a bDOT only captures administration of the antibiotic if the provider also billed on that same date, thus excluding DOT for the patient if cared for by a different service team (care escalated to ICU, transfer to surgery service). To streamline the focus and eliminate the need to track specific antibiotics, all bDOTs were summed by NHSN group for each provider. If three separate antibiotic agents belonging to the same NHSN group had the same transaction date for the same provider, this equated to three bDOTs for that NHSN group on that date for the provider.

### Patient encounter characteristics

Second, we extracted patient characteristics linked to each encounter, including age, antibiotic indications (pneumonia, urinary tract infection [UTI]), and comorbidities/disease severity using ICD-10 codes. These included sepsis, obesity, COVID-19, end-stage renal disease (ESRD), neurological conditions, sepsis, malignancies, and the Charlson Comorbidity Index (CCI). These characteristics were identified a priori as patient factors that were potential predictors of antibiotic prescribing in hospital settings.^19^

### Exclusions, attribution, and aggregation parameters

Based on feedback from Division of Hospital Medicine Leadership regarding this initiative, as well as a series of focus groups with volunteer Hospital Medicine Providers, we defined some parameters for eligible data for inclusion into a summary metric. These parameters included exclusion of data related to patients with cystic fibrosis (roughly 5% of encounters) from the analysis since antibiotic prescriptions for this subset were determined by pulmonary clinicians, not hospitalists. We also excluded data on hospitalists who worked nights exclusively (i.e., 8 nocturnists) since the data captured were largely reflective of prescribing practices of other service lines such as emergency medicine and critical care. Over the study period 12 Advanced Practice Providers worked with a variety of Faculty, and prescribing of Advanced Practice Providers (APPs) were attributed to the Faculty billing for the patient care that day. All measures (bPD, patient characteristics, and bDOT) were aggregated into bimonthly (every two months) intervals, resulting in provider-level datasets stratified by hospital. Next, provider-specific data for any single bimonthly period was excluded from analysis if the provider accrued fewer than 80 bPD, suggested they worked < 2 weeks during the preceding month. The bimonthly metric was the consensus aggregation level to allow providers more regular feedback in a period they still could recollect their patient care experience. This approach was adopted to reduce data sparsity for providers with variable work schedules reflective in a monthly metric. Finally, both Hospital Medicine Service (HMS) leadership and the HMS focus groups universally agreed they would prefer hospital-specific risk adjustments (i.e., facility-specific models as described below) due to the drastically different nature of patient mix between the four facilities (as reflected in Table 1).

**Table 1.** Summary of billed patient-days and patient-mix characteristics for hospital medicine providers at four hospitals within an academic healthcare network in Georgia (2020–2021)

	Hospital A	Hospital B	Hospital C	Hospital D
Total Bimonthly Observations	282	286	225	138
Hospital Medicine Providers	43	37	32	17
Bimonthly Observations per Provider, n (%)				
1	2 (4.7)	1 (2.7)	2 (6.2)	0 (0)
2-8	19 (44.2)	14 (37.8)	12 (37.5)	3 (17.6)
9	22 (51.2)	22 (59.5)	18 (56.2)	14 (82.4)
Billed patient-days (bPD)				
Total	88,444	89,537	74,117	40,937
Bimonthly, Median (Q1, Q3)	336 (238, 393)	326 (227, 389)	357 (220, 431)	315 (226, 354)
% bPD (Median [Q1, Q3]) attributed to care for patients with				
Age > 65 years	43.4 (38.4, 47.9)	42.1 (35.0, 46.7)	59.8 (56.2, 64.5)	53.7 (48.1, 57.9)
Obesity	37.2 (33.3, 41.5)	36.5 (32.7, 40.5)	33.6 (29.7, 36.9)	33.4 (29.3, 37.2)
Pneumonia	29.5 (22.7, 38.5)	23.4 (18.1, 28.0)	24.3 (18.1, 33.0)	23.2 (18.7, 32.4)
COVID19	10.0 (3.3, 21.7)	12.7 (7.0, 19.8)	11.2 (3.8, 21.6)	6.5 (2.3, 18.5)
Sepsis	24.7 (19.7, 30.9)	20.3 (16.8, 24.9)	25.2 (20.5, 28.9)	19.2 (16.2, 22.7)
UTI	18.7 (14.5, 23.0)	13.4 (10.1, 17.7)	18.4 (15.7, 21.1)	17.6 (13.7, 21.5)
CC1>2	58.9 (54.1, 64.0)	61.3 (54.2, 67.5)	57.1 (52.6, 62.1)	47.6 (43.9, 52.8)
Malignancy	11.8 (9.3, 14.8)	16.7 (13.2, 20.1)	14.8 (11.4, 17.2)	14.9 (11.3, 19.7)
Neurological disorder	68.5 (62.8, 74.6)	60.4 (55.2, 64.9)	59.4 (55.3, 64.4)	56.4 (52.9, 60.2)
ESRD	10.3 (7.5, 13.0)	8.3 (3.7, 24.3)	9.0 (6.9, 11.3)	5.0 (3.1, 6.8)
Antibiotic Days of Therapy (DOT), Bimonthly Median (Q1, Q3)				
BSHO	16 (8, 25)	9 (5, 15)	26 (16, 35)	13 (7, 17)
BSCA	19 (10, 29)	15 (9, 23)	23 (15, 31)	23 (15, 34)
Anti-MRSA	16 (8, 23)	11 (6, 17)	12 (7, 20)	9 (5, 14)
Observed Prescribing (DOT/1000bPD), Bimonthly Median (Q1-Q3)				
BSHO	51.6 (30.0, 74.0)	30.2 (16.0, 44.5)	79.1 (59.0, 95.1)	43.7 (28.7, 54.5)
BSCA	62.1 (36.8, 82.7)	47.7 (34.8, 65.1)	69.1 (55.1, 84.9)	77.7 (59.7, 105.7)
Anti-MRSA	49.3 (32.5, 65.9)	37.1 (23.0, 51.8)	39.1 (26.4, 49.8)	32.6 (16.6, 47.3)

BSHO, Broad-spectrum hospital-onset antibiotics; BSCA, Broad-spectrum community-acquired antibiotics; Anti-MRSA, Anti-Methicillin-resistant Staph, Aureus antibiotics; ESRD, End-Stage Renal Disease; UTI, Urinary tract Infection; CCI, Charlson Comorbidity Index.

### Observed-to-expected ratios (OERs)

The main outcome was provider-specific observed-to-expected ratios (OERs), a metric quantifying the deviation of observed prescribing from the expected, given the characteristics of patients seen by the provider within the specified period. OERs were calculated by dividing observed prescribing by the expected prescribing derived using methods detailed below.

### Statistical analysis

The prescribing characteristics of the providers, total bPD, and bDOT for the three NHSN groups were presented as medians and interquartile ranges (IQR). The proportions of the bimonthly bPD attributed to care for patients with each patient-mix characteristic were calculated for each provider and described for each hospital using medians and IQR. For each provider, the observed prescribing of each NHSN antibiotic group was calculated for each bimonthly interval as a ratio of the observed bDOT for that antibiotic group and the total bPD within the interval. These data were described across hospitals using medians and interquartile ranges (IQR).

Expected prescribing was estimated using patient-mix adjusted linear mixed regression models with random intercepts to account for repeated measurements of provider prescribing over time. The expected prescribing predictive model selection process involved two stages. First, we employed backward elimination in linear mixed models across 1000 bootstrapped samples to identify variable inclusion frequencies and establish candidate models.^20,21^ Bootstrap samples were created by sampling at the provider-level with replacement and included all bimonthly observations associated with selected providers in the bootstrap sample.^22,23^ Providers selected more than once in a bootstrap sample were assigned different subject-level identifiers to ensure treatment within the model as distinct subject clusters. A linear mixed-effects model, adjusted for all ten patient characteristics measured, was fitted to each bootstrap sample, and statistically significant covariates (alpha = 0.05) were identified using backward elimination (fixed effects only). The frequency of each covariate being selected as a significant predictor of antibiotic prescribing across all bootstrap samples used was calculated and ranked. Ten candidate models were then created by sequentially adding these potential predictors to a mean model, starting with the highest-ranked predictor.

### Model comparisons

The predictive accuracy of the candidate models was quantified and ranked using the bootstrap-based optimism-correction method.^24,25^ This method was selected due to the small number of hospitalists and/or bimonthly observations (number of unique hospital medicine providers per hospital <50; number of bimonthly observations per hospital <300). First described by Harrell et al, the bootstrap-based optimism-correction method is an internal predictive model validation method that involves repeatedly sampling from a study sample to measure and correct for anticipated optimism from model overfitting.^24–26^ This approach provides an alternative way of internally validating predictive models for small samples where more robust model validation methods that require data splitting may not be prudent and has been shown to perform similarly to other model validation methods.^24,26^ We estimated optimism-corrected mean square errors (OC-MSE) for each candidate model (CM_k_) by fitting the model to the hospital dataset (D) and calculating the mean square error (MSE), C (step 1). Then, in a bootstrap sample of the hospital dataset (D*), we refitted CM_k_ and calculated the MSE (C_boot_, step 2). Using the coefficients of model fit in D*, we predicted outcomes in the original dataset, D, and re-evaluated the MSE (C_orig_, step 3). The optimism was then calculated as C_boot_-C_orig_ (step 4). Steps 2 through 4 were repeated 200 times and the average optimism (O) was calculated (step 5). For each CM_k_, OC-MSE was calculated as C – O (step 6). The OC-MSEs for all 10 candidate models were then ranked and the model with the lowest OC-MSE was selected as the final prediction model. These final risk-adjusted models were fitted to original hospital datasets and used to estimate expected prescribing adjusted for patient-mix characteristics for each provider in each bimonthly interval. Serial provider-specific OERs were then calculated as the ratio of observed prescribing to expected prescribing in each bimonthly interval.

### Sensitivity analysis

We evaluated expected prescribing predictive model selection, as described above, against information criterion-based model selection. This was done by assessing agreement in model selections across OC-MSE, marginal Akaike information criterion (mAIC), and conditional Akaike information criterion (cAIC) methods.^27–29^ For each NHSN group and hospital, we derived mAIC and cAIC for all candidate models considered and identified the models that most minimized each information criterion.^29,30^ Then, we assessed how frequently the OC-MSE selected models matched the final models selected using the information criterion (absolute agreement). We also assessed how frequently the information criteria estimates of the OC-MSE selected models were within +/–2 of the lowest information criteria estimated for the group of candidate models (relative agreement). All analyses were performed using R Statistical Software (v4.2.0; R Core Team 2021).

## Results

### Study sample description

**Figure 1.**
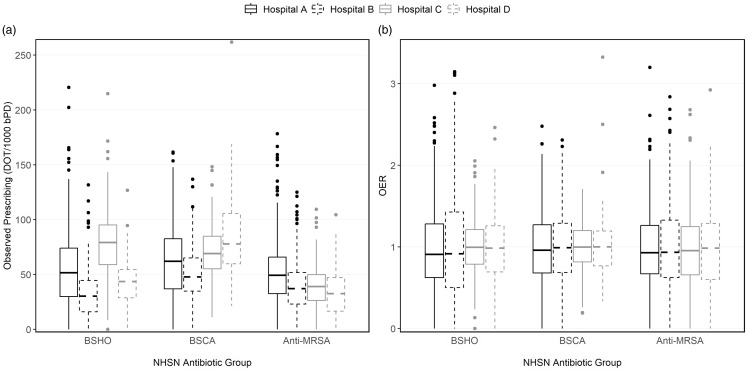
Variations in observed prescribing (panel a) and observed-to-expected ratios (panel b) across four hospitals in an academic healthcare network, Georgia (2020-2021). BSHO, Broad-spectrum hospital-onset antibiotics; BSCA, Broad-spectrum community-acquired antibiotics; Anti-MRSA, Anti-Methicillin-resistant Staph. Aureus antibiotics.

Across the four hospitals, 136 hospital medicine providers contributed to 975 bimonthly observations during the 18-month study period (Table 1). The number of observations per hospitalist ranged from 1 to 9, with over 50% of providers included in the study contributing data for all nine-time points. The total bPD reported exceeded 300,000 across all hospitals, with notable variations in patient-mix characteristics. Community hospitals (Hospital C: 60%, Hospital D: 53%) had a greater proportion of bPD attributed to care for older patients (65 years and older) than did the academic hospitals (Hospital A: 43%, Hospital B: 42%). Larger hospitals (Hospitals A, B, and C) also had greater proportions of overall bPD attributed to care for patients with ESRD and CCI >2 designations than did the smallest hospital (Hospital D). The observed prescriptions varied across hospitals (Figure 1a). Hospital C exceeded other hospitals in use of BSHO antibiotics, with hospitalists prescribing a median of 79.1 (IQR: 59.0, 95.1) BSHO DOT per 1000 bPD—more than twice the median BSHO DOT recorded at Hospital B (30.2 [IQR: 16.0, 44.5]). BSCA antibiotics were most commonly prescribed at Hospital D (77.7 [IQR: 57.9, 105.7]), while Anti-MRSA antibiotics were most commonly prescribed at Hospital A (49.3 [IQR: 32.5, 65.9]).

### Risk-adjustment models and patient-mix predictors of antibiotic prescribing among hospitalists

For each hospital, three antibiotic-prescribing predictive models, corresponding to each NHSN antibiotic group, were identified (Table 2). Various combinations of patient-mix characteristics predicted antibiotic prescribing across the four hospitals (Table 2 and Supplemental Table S2). Sepsis emerged as the most frequently selected predictor, featured in 8 (66.7%) of the 12 final models. Other commonly selected patient characteristics included COVID-19 (50%) and pneumonia (50%) diagnoses, UTI (42%), older age (42%), and malignancy (42%). As expected, diagnoses of bacterial infections (sepsis, UTI, pneumonia), older age, obesity, and CCI >2 (indicating moderate-to-severe comorbidity) were largely positive predictors of antibiotic prescribing, while COVID-19 diagnoses, neurological disorders, and malignancy were predominantly negative predictors. Sensitivity analysis revealed substantial agreement in the final model composition when OC-MSE-based selections were compared to information criterion-based final model selections. Complete agreement in the final model compositions occurred for 6 (50%) and 8 (67%) of the 12 final models when mAIC and cAIC were used as criteria for selection instead of OC-MSE. However, there was 92% relative agreement, with the OC-MSE-selected model being within a 5-point range of the mAIC- or cAIC-selected model for 11 of 12 antibiotic-prescribing predictive models.

**Table 2.** Patient-mix characteristics predicting NHSN antibiotic prescribing among hospital medicine providers in four hospitals in an academic healthcare network, Georgia (2020–2021)

	Hospital A			Hospital B			Hospital C			Hospital D			
	BSHO	BSCA	Anti-MRSA	BSHO	BSCA	Anti-MRSA	BSHO	BSCA	Anti-MRSA	BSHO	BSCA	Anti-MRSA	Selected^a^ n (%)
Sepsis	+	+	+	+		+	+	–	+				8 (66.7)
COVID-19		+	–				–	–			–	–	6 (50.0)
Pneumonia		+			+	–	–	+			+		6 (50.0)
Age > 65 years	+	+	+				+				+		5 (41.7)
Malignancy			+				+			–	–	–	5 (41.7)
UTI		+			+			+		+	+		5 (41.7)
Neurological Disorders			–			+	–	–					4 (33.3)
ERSD			–	+						+			3 (25.0)
Obesity			–	+							+		3 (25.0)
CCI > 2						+						+	2 (16.7)

“+” = Positive predictor of antibiotic prescribing among Hospital Medicine Providers (see coefficients in Supplemental Table S1).

“−” = Negative predictor of antibiotic prescribing among Hospital Medicine Providers (see coefficients in Supplemental Table S1).

BSHO, Broad-spectrum hospital-onset antibiotics; BSCA, Broad-spectrum community-acquired antibiotics; Anti-MRSA, Anti-Methicillin-resistant Staph. Aureus antibiotics; ESRD, End-Stage Renal Disease; UTI, Urinary tract infection; CCI, Charlson Comorbidity Index

a Number of times characteristic was selected as a predictor of NHSN antibiotic prescribing in a final predictive model (model selections made based on optimism-corrected MSE).

### Observed-to-expected prescribing ratios and patterns of high prescribing at the four hospitals

**Figure 2.**
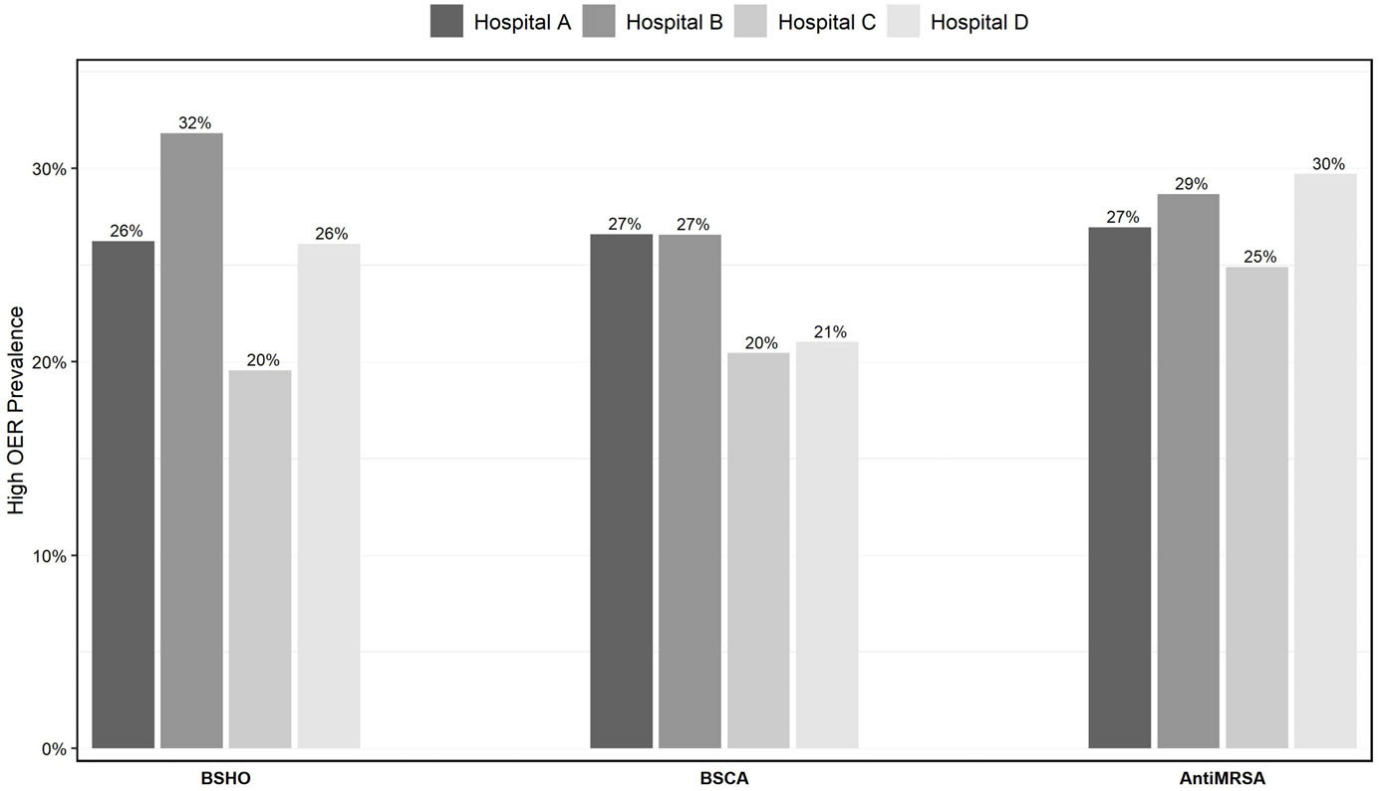
Prevalence of high antibiotic prescribing (observed-to-expected ratio > 1.25) prevalence at four hospitals in an academic healthcare network, Georgia (2020-2021). BSHO, Broad-spectrum hospital-onset antibiotics; BSCA, Broad-spectrum community-acquired antibiotics; Anti-MRSA, Anti-Methicillin-resistant Staph. Aureus antibiotics.

The median OER was approximately 1 across all hospitals and antibiotic groupings (Figure 1b and Supplemental Table S2). However, within each hospital, substantial variability in OERs was evident, with the 75th percentile of estimated OERs at approximately 1.25 and a maximum that exceeded 3.30 (Figure 1b). Using 1.25 (the 75th percentile) as OER cutoff for high prescribing, hospital medicine providers across the four hospitals were found to prescribe more antibiotics than expected in approximately one-quarter of the observed time points on average (Figure 2). Any one provider’s OER varied substantially between sequential periods (Figure S1) although the overall percent of providers reporting a high OER varied only slightly between periods (Figure S2). There were only modest shifts in any one provider’s relative rank using crude rates compared to adjusted OER, with only 3–10 providers shifting for highest or lowest quartile to another quartile in any bimonthly period after applying the risk adjustment across all facilities (roughly 5% of providers in any facility).

## Discussion

In this multicenter study, we established a patient risk-adjusted metric suitable for monitoring antibiotic use among hospitalists. Presently, the CDC’s tracking system focuses on aggregate usage rates at the location or hospital level, lacking attribution to individual providers. Although aggregated values may align with specific providers in certain healthcare settings, this is not the norm in most U.S. acute care hospitals. We demonstrate a process that employs prescribing attribution to the dates when a provider cared for specific patients, curating a personalized metric more likely to better reflect a specific provider’s behavior and serve as a clinically credible tool for performance improvement.

We observed a median OER of 1 across all antibiotic groups and hospitals, illustrating that this metric can be used to standardize a metric across facilities caring for a diverse patient populations in an integrated system and can serve as a baseline for measuring antibiotic use. However, the considerable variability in OERs within each hospital highlights potential differences in prescribing behaviors between providers. This variability may reflect variations in adherence to antibiotic stewardship guidelines among providers, necessitating further investigation into factors contributing to both over- and under-prescribing. Understanding these sources of variability is essential for developing targeted interventions to optimize antibiotic prescribing practices, particularly among providers who consistently produce high O:E values, and to enhance overall stewardship efforts.

We believe that models such as ours hold great potential as standardized benchmarks for monitoring usage trends, as demonstrated by the observed changes in the OER over time. The fluctuations over time may correspond to shifts in treatment indications such as the sudden fluctuations in OERs observed at two facilities during the peak of the COVID-19 pandemic or as we believe in the case of anti-MRSA therapy, antibiotic stewardship interventions. Notably, we observed consistent downward trends in the median OER for the Anti-MRSA antibiotic groups at two hospitals, aligning with a practice change initiated by the stewardship team to reduce unnecessary anti-MRSA therapy by adopting use of a rapid screening test to detect nasal colonization with MRSA, which was introduced during the early months of the study period.

This study has several limitations. A very important limitation is the presence of unmeasured confounders related to prescriber habits or influences, such as infectious disease consultation, which were accounted for in the predictive modeling. Other limitations relate to the analytic approach. The relationship between antibiotic prescribing and patient-mix characteristics across the study sites was assumed to be linear, and the outcome, expected antibiotic prescribing, was modeled using linear mixed-effects regression. Although a generalized Poisson model might have been more ideal, linear mixed models were chosen to mitigate issues with model convergence and overdispersion during bootstrapping, given the complexity of the method used. Another limitation is the use of Harrell’s optimism-correction method for internal validation of the prediction models in OER derivation. While this method is comparable to more robust approaches like the cross-validation method, it may exhibit an overly optimistic bias, potentially overestimating the predictive performance.^31^ Our sensitivity analysis, however, indicates that the final models selected were reasonably comparable to those that might have been selective if alternative model selection methods were used. Future studies could benefit from using larger sample sizes, which would enable the use of more robust internal validation methods that allow data splitting and incorporation of external validation of selected models.^32^ Related as well to sample size, our bimonthly metric was imprecise, each OER had wide confidence intervals (data not show), however we used bimonthly values because of the providers preference to have more real-time estimates rather than more precise yearly values. Finally, low R-squared values for all 12 final models suggested that the predictors used in the models explained less than an ideal proportion of the total variability in the study outcomes, possibly due to the limited array of predictors included in the analysis. Perhaps as a result of poor model fitting, few prescribers moved substantially from one relative quartile of OER values to another quartile.

We believe these data support the exploration of risk-adjusted prescribing metrics over crude metrics in clinical trials or quality improvement activity using provider-specific despite these limitations. There are likely other patient characteristics that may better predict antibiotic prescribing better that were not measured in this study. However, the extensive residual variability in the prescribing metric suggests that factors unique to provider and provider-patient interactions likely influenced prescribing habits. Future efforts should include refinement accounting for any influence of consultation by Infectious Disease Providers; however immediate next steps exploring the impact of peer-comparisons of prescribing metrics among inpatient providers may be needed to justify further advancements in this emerging stewardship tool for inpatient settings.

## Supporting information

Onwubiko et al. supplementary material Onwubiko et al. supplementary material

## Supplementary material

For supplementary material accompanying this paper visit http://doi.org/10.1017/ash.2024.422. click here to view supplementary material

## Research transparency and Reproducibility

The datasets generated and/or analyzed for the current study are to be available in the Emory Dataverse repository (URL: https://dataverse.unc.edu/dataverse/Emory). To eliminate risk of identification, hospital facility characteristics (bed size, location, etc.) will be suppressed to minimize identification of institution. All personally identifying information (e.g., names, addresses, phone numbers, etc.) is absent from the data in compliance with Department of Health and Human Services Guidance Regarding Methods for De-identification of Protected Health Information in Accordance with the Privacy Rule to implement the requirement of the Health Insurance Portability and Accountability Act of 1996.
