# Supplementary material for: Derivation of a risk-adjusted model to predict antibiotic prescribing among hospitalists in an academic healthcare network
Source: Antimicrob Steward Healthc Epidemiol. 2024 Oct 7;4(1):e163. doi: 10.1017/ash.2024.422 (PMC11474874; doi:10.1017/ash.2024.422)
Supplement: Onwubiko et al. supplementary material [file S2732494X24004224sup001.docx]

Supplemental Table S1: Participating Hospitals Characteristics and Patient population distribution

| Hospital | Characteristics | Bed Count | % African American | % Hispanics | % Without a Primary care physician | % Uninsured |
| --- | --- | --- | --- | --- | --- | --- |
| Hospital A | Suburban, non-profit | 582 | 46.1% | 5.2% | 40.1% | 4.7% |
| Hospital B | Urban, non-profit | 537 | 71.6% | 3.1% | 53.6% | 6.4% |
| Hospital C | Suburban, non-profit | 373 | 32.4% | 5.8% | 43.7% | 3.9% |
| Hospital D | Suburban, non-profit | 152 | 19.7% | 6.7% | 36.8% | 4.4% |

Supplemental Table S2: Beta Coefficients, 95% Confidence Intervals and P-values for Risk-Adjusted Models Predicting NHSN Antibiotic Prescribing Among Hospital Medicine Providers in Four Hospitals within an Academic Healthcare Network, Georgia (2020-2021)

|  | **BSHO** | |  | **BSCA** | |  | **Anti-MRSA** | |
| --- | --- | --- | --- | --- | --- | --- | --- | --- |
|  | **β coefficient (95% CI)** | **p-value** |  | **β coefficient (95% CI)** | **p-value** |  | **β coefficient (95% CI)** | **p-value** |
| **Hospital A** | | | | | | | | |
| Age > 65 yrs. | 0.59 (0.08, 1.09) | 0.024 |  | 0.34 (-0.17, 0.85) | 0.187 |  | 0.47 (-0.01, 0.96) | 0.057 |
| Obesity |  |  |  |  |  |  | -0.23 (-0.79, 0.32) | 0.415 |
| UTI |  |  |  | 0.79 (0.22, 1.37) | 0.007 |  |  |  |
| Pneumonia |  |  |  | 0.33 (-0.20, 0.87) | 0.223 |  |  |  |
| COVID19 |  |  |  | 0.35 (-0.07, 0.77) | 0.107 |  | -0.19 (-0.45, 0.08) | 0.161 |
| Sepsis | 1.63 (1.13, 2.14) | <0.0001 |  | 0.93 (0.42, 1.45) | <0.001 |  | 1.31 (0.80, 1.82) | <0.0001 |
| CCI>2 |  |  |  |  |  |  |  |  |
| Malignancy |  |  |  |  |  |  | 0.45 (-0.38, 1.28) | 0.285 |
| Neurological Disorders |  |  |  |  |  |  | -0.42 (-0.81, -0.02) | 0.038 |
| ERSD |  |  |  |  |  |  | -0.46 (-1.15, 0.22) | 0.187 |
| **Hospital B** | | | | | | | | |
| Age > 65 yrs. |  |  |  |  |  |  |  |  |
| Obesity | 0.34 (-0.06, 0.74) | 0.095 |  |  |  |  |  |  |
| UTI |  |  |  | 0.89 (0.33, 1.44) | 0.002 |  |  |  |
| Pneumonia |  |  |  | 0.36 (-0.02, 0.75) | 0.062 |  | -0.37 (-0.73, -0.01) | 0.046 |
| COVID19 |  |  |  |  |  |  |  |  |
| Sepsis | 0.43 (0.00, 0.86) | 0.049 |  |  |  |  | 0.91 (0.41, 1.41) | <0.001 |
| CCI>2 |  |  |  |  |  |  | 0.18 (-0.10, 0.45) | 0.208 |
| Malignancy |  |  |  |  |  |  |  |  |
| Neurological Disorders |  |  |  |  |  |  | 0.35 (-0.03, 0.73) | 0.074 |
| ERSD | 0.35 (0.20, 0.51) | <0.0001 |  |  |  |  |  |  |
| **Hospital C** | | | | | | | | |
| Age > 65 yrs. | 0.64 (0.02, 1.26) | 0.043 |  |  |  |  |  |  |
| Obesity |  |  |  |  |  |  |  |  |
| UTI |  |  |  | 2.14 (1.49, 2.80) | <0.0001 |  |  |  |
| Pneumonia | -0.17 (-0.89, 0.56) | 0.646 |  | 0.92 (0.37, 1.46) | 0.001 |  |  |  |
| COVID19 | -0.39 (-0.90, 0.13) | 0.142 |  | -0.53 (-0.92, -0.14) | 0.007 |  |  |  |
| Sepsis | 0.91 (0.19, 1.63) | 0.013 |  | -0.58 (-1.14, -0.02) | 0.041 |  | 0.53 (0.13, 0.92) | 0.010 |
| CCI>2 |  |  |  |  |  |  |  |  |
| Malignancy | 0.87 (-0.08, 1.81) | 0.072 |  |  |  |  |  |  |
| Neurological Disorders | -0.58 (-1.20, 0.05) | 0.070 |  | -0.44 (-0.90, 0.03) | 0.066 |  |  |  |
| ERSD |  |  |  |  |  |  |  |  |
| **Hospital D** | | | | | | | | |
| Age > 65 yrs. |  |  |  | 0.49 (-0.28, 1.26) | 0.211 |  |  |  |
| Obesity |  |  |  | 1.31 (0.40, 2.23) | 0.005 |  |  |  |
| UTI | 0.96 (0.33, 1.59) | 0.003 |  | 2.15 (1.15, 3.16) | <0.0001 |  |  |  |
| Pneumonia |  |  |  | 1.75 (0.70, 2.79) | 0.001 |  |  |  |
| COVID19 |  |  |  | -1.09 (-1.89, -0.30) | 0.007 |  | -0.38 (-0.63, -0.13) | 0.003 |
| Sepsis |  |  |  |  |  |  |  |  |
| CCI>2 |  |  |  |  |  |  | 0.68 (0.15, 1.20) | 0.012 |
| Malignancy | -0.65 (-1.15, -0.15) | 0.011 |  | -1.60 (-2.39, -0.82) | <0.001 |  | -1.21 (-1.73, -0.68) | <0.0001 |
| Neurological Disorders |  |  |  |  |  |  |  |  |
| ERSD | 1.58 (0.22, 2.95) | 0.023 |  |  |  |  |  |  |

**Abbreviations: BSHO** = Broad-spectrum hospital onset antibiotics; **BSCA** = Broad-spectrum community acquired Antibiotics; **Anti-MRSA** = Anti-Methicillin-resistant Staph. Aureus antibiotics; **ESRD** = End-Stage Renal Disease; **UTI** = Urinary tract Infection; **CCI** = Charlson Comorbidity Index

Supplemental Table S3: OER Distribution in Four Hospitals within an Academic Healthcare Network, Georgia (2020-2021)

| **NHSN Antibiotic Group** | **Hospital A** | **Hospital B** | **Hospital C** | **Hospital D** |
| --- | --- | --- | --- | --- |
| Total Bimonthly Observations | 282 | 286 | 225 | 138 |
| Hospital Medicine Providers | 43 | 37 | 32 | 17 |
|  |  |  |  |  |
| **BSHO** |  |  |  |  |
| Median (Q1, Q3) | 0.91 (0.62, 1.28) | 0.92 (0.50, 1.43) | 1.00 (0.79, 1.21) | 0.99 (0.69, 1.26) |
| Min. – Max. | 0.00 - 2.98 | 0.00 - 3.15 | 0.00 - 2.05 | 0.00 - 2.46 |
| **BSCA** |  |  |  |  |
| Median (Q1, Q3) | 0.96 (0.68, 1.27) | 0.99 (0.69, 1.29) | 1.00 (0.82, 1.20) | 1.00 (0.77, 1.20) |
| Min. – Max. | 0.00 - 2.48 | 0.00 - 2.31 | 0.19 - 1.71 | 0.33 - 3.32 |
| **Anti-MRSA** |  |  |  |  |
| Median (Q1, Q3) | 0.93 (0.67, 1.26) | 0.93 (0.63, 1.33) | 0.96 (0.66, 1.25) | 0.99 (0.60, 1.29) |
| Min. – Max. | 0.00 - 3.20 | 0.00 - 2.84 | 0.00 - 2.68 | 0.00 - 2.92 |

Abbreviations**: BSHO** = Broad-spectrum hospital onset antibiotics; **BSCA** = Broad-spectrum community acquired Antibiotics; **Anti-MRSA** = Anti-Methicillin-resistant Staph. Aureus antibiotics

**Supplemental Figure S1. Trends in Provider-specific OERs and Median OER for Antibiotic Prescribing at Four Hospitals in an Academic Healthcare Network, Georgia (2020-2021).** Grey lines represent the provider-specific OERs. Thick black line represents the median OER at each hospitality. BSHO = Broad-spectrum hospital onset antibiotics; BSCA = Broad-spectrum community acquired Antibiotics; Anti-MRSA = Anti-Methicillin-resistant Staph. Aureus antibiotics

**Supplemental Figure S2: Trends in High Prescribing (OER > 1.25) Prevalence Among Hospital Medicine Providers at Four Hospitals in an Academic Healthcare Network, Georgia (2020-2021).** BSHO = Broad-spectrum hospital onset antibiotics; BSCA = Broad-spectrum community acquired Antibiotics; Anti-MRSA = Anti-Methicillin-resistant Staph. Aureus antibiotics
